# A high-density genome-wide approach reveals novel genetic markers linked to small ruminant lentivirus susceptibility in sheep

**DOI:** 10.3389/fgene.2024.1376883

**Published:** 2024-06-06

**Authors:** Silvia Riggio, Marco Tolone, Gianluca Sottile, Serena Tumino, Baldassare Portolano, Anna Maria Sutera, Maria Teresa Sardina, Alberto Cesarani, Salvatore Mastrangelo

**Affiliations:** ^1^ Dipartimento Scienze Agrarie, Alimentari e Forestali, University of Palermo, Palermo, Italy; ^2^ Dipartimento di Scienze Chimiche, Biologiche, Farmaceutiche, ed Ambientali, University of Messina, Messina, Italy; ^3^ Dipartimento Scienze Economiche, Aziendali e Statistiche, University of Palermo, Palermo, Italy; ^4^ Dipartimento di Agricoltura, Alimentazione e Ambiente, University of Catania, Catania, Italy; ^5^ Dipartimento di Agraria, University of Sassari, Sassari, Italy; ^6^ Department of Animal and Dairy Science, University of Georgia, Athens, GA, United States

**Keywords:** small ruminant lentiviruses, local sheep, genome-wide approaches, high density array, candidate genes

## Abstract

Visna/Maedi virus (VMV) is lentiviral disease of sheep responsible for severe production losses. Multiple genomic regions associated with infection were reported indicating genetic complexity. In this study, a combined genome-wide approach using a high-density SNP array has been performed, comparing VMV-infected (*n* = 78) and non-infected (*n* = 66) individuals of the Valle del Belice breed. The serological tests showed a seroprevalence of 26%. The comparison among results from different approaches (GWAS, Fisher’s exact test and the F_ST_ analysis) revealed two association signals: on OAR03 close to the *GRIN2B* gene and on OAR05 close to the *TMEM232* gene. To the best of our knowledge, there has been no previous association between these genes and lentiviral infection in any species. The *GRIN2B* gene plays a role in pain response, synaptic transmission, and receptor clustering, while *TMEM232* is involved in the development of immune-related disorders. The results highlighted new aspects of the genetic complexity related to the resistance/susceptibility to VMV in sheep, confirming that studies on different breeds can lead to different results. The ideal approach for validation of the markers identified in our study is to use samples from a population independent from the discovery population with the same phenotype used in the discovery stage.

## 1 Introduction

Sheep and goats are infected by Visna/Maedi virus (VMV) and caprine arthritis encephalitis virus (CAEV) (respectively), which belong to the *retroviridae* family and are classified as small ruminant lentiviruses (SRLV). According to Minguijón et al. (2015), a wasting disease marked by persistent inflammation in carpal joints, udder, central nervous system, and lungs is caused by these viruses. The main productive and economic losses associated to the diseases are reduced milk production and ewe productivity, lower birth weights in offspring, early culling or death of the infected animals, cost of the control programs and export restrictions (Peterhans et al., 2004; Benavides et al., 2013; Gibson et al., 2018; Juste et al., 2020). The transmission of the disease can occur either through the ingestion of colostrum from infected ewes or through direct contact with the respiratory secretions of infected animals (Preziuso et al., 2004; Kalogianni et al., 2020). The VM shows a long incubation period, which is reported to be around three or 4 years, and a slow progressive and subclinical infection (Zink et al., 1987; Straub, 2004). Moreover, the slow seroconversion in the infected animals leads to an underdiagnosed and underreported disease rate and to high frequency of asymptomatic carriers (Illius et al., 2020).

Immune responses to infection result in antibodies that are typically detected throughout the entire life of the animals. Therefore, since there is no vaccination for SRLV, the control of this disease mainly relies on identifying seropositive animals (Kalogianni et al., 2020). Multiple diagnostic techniques can be used to detect SRLV infection and indirect methods have been proposed as the most appropriate to detect infected animals. In fact, the association between breeds and SRLV infection susceptibility patterns has been generally discovered using ELISA testing for the antibody’s detection (de Miguel et al., 2021).

In viral infections, without therapies or efficient vaccinations, genetic selection of resistant animals is of special interest (de Pablo-Maiso et al., 2018). The genome-wide approaches based on the Single Nucleotide Polymorphisms (SNPs) arrays created new opportunities to include disease control in the breeding programs. In particular, the genetic tools have been used to describe several candidate genes associated with SRLV seroreaction (Abendaño et al., 2021). The *TMEM154* has been reported to be the major gene associated to VMV susceptibility by Heaton et al. (2012) and several subsequent independent studies (Molaee et al., 2018; Molaee et al., 2019; Yaman et al., 2019; Ramírez et al., 2021; Tumino et al., 2022; Riggio et al., 2023; Rodrigues et al., 2023). The polymorphism involved in resistance/susceptibility is located in the portion of the gene encoding the extracellular part of the protein and is characterised by the substitution, at position 35 of the protein, of glutamate (E) amino acid with lysine (K) one (Heaton et al., 2012). The resistance occurred only in homozygous KK animals, while heterozygous EK and homozygous EE were susceptible to disease. However, a genetic complexity of this disease can be supposed because of the several candidate genes associated with the resistance/susceptibility to VMV (Abendaño et al., 2021; Arcangeli et al., 2021), such as: *CCR5* (White et al., 2009; Molaee et al., 2018), *ovar-DRB1* (Larruskain et al., 2010), *DPPA2/DPPA4* and *SYTL3* (White et al., 2012), *TLR9* and *MYD88* (Sarafidou et al., 2013) and *ZNF389* (White et al., 2014). Additionally, the disease can manifest differently depending on management systems, VMV strains, age, immune status, and sex of animals; furthermore, much evidence showed that susceptibility to SRLV infection exhibits significant variation across different breeds, contributing to the complexity of the trait (e.g., Keen et al., 1997; Molaee et al., 2019; Letko et al., 2021; Moretti et al., 2022).

To date, few sheep GWAS studies aimed to identify additional potentially candidate genes involved in the resistance to VMV. On these premises, in this study, genome-wide approaches have been used to compare VMV-infected and non-infected genotyped individuals of the Valle del Belice sheep breed to identify candidate genomic regions potentially involved with the resistance to VMV infection.

## 2 Materials and methods

### 2.1 Animals and samples

Before collecting samples, a preliminary screening was carried out on 20 farms, to assess the prevalence status of Visna/Maedi in the Valle del Belìce breed within different herds. After this, the sampling was carried out in eight farms, in which the prevalence status of Visna/Maedi was at least 20%. Therefore, samples were collected from sheep flocks already known to be VM affected.

Following the recommendations of the European Union Directive 2010/63/EU, individual blood samples were collected from 300 ewes. The animals did not show clinical signs of the disease. From all involved ewes, an individual milk sample was also collected during the morning milking. All the farms raised only sheep and shared a similar management based on daytime grazing and night shelter. The epidemiological unrelatedness of farm was ensured by the lack of animal transfer among flocks. To allow for sufficient VMV exposure and subsequent seroconversion, age of sampled ewes was from 2 to 8 years (mean 4.42 ± 1.33).

### 2.2 Serological analyses by ELISA test

The milk samples were analyzed by the commercial ELISA assay ID Screen^®^ MVV/CAEV Indirect Screening test (IDvet, Grabels, France), following the official instructions from the manufacturer. As the final phase of the process, ELISA plates were read at 450 nm wavelength using an ELISA plate reader. The cut-off value was defined based on the corrected optical density (OD) ratio between sample and positive control (S/P) at a wavelength of 450 nm. As suggested in the instructions provided by the manufacturer, samples were considered SRLVs negative (and defined control) or positive (and defined case) with S/*p*-value ≤ 50% or ≥60%, respectively.

To exclude false positives within samples, serological positive animals were tested twice using the ID Screen^®^ MVV/CAEV. This retesting occurred at two distinct time points: first, during the initial screening to assess Visna/Maedi prevalence across all herds, and again 6 months later for the animals specifically chosen for the study.

### 2.3 DNA extraction, genotyping and quality control

Following the protocol of the PureLink Genomic DNA kit (Invitrogen), genomic DNA was extracted from 200 μL of blood. The DNA was quantified using the Nanodrop ND-1000 (Thermo Fisher Scientific).

All the individuals were genotyped at the *TMEM154* gene by direct Sanger sequencing, to estimate the genotype frequencies of the E35K polymorphism (Riggio et al., 2023).

A subset of 144 animals including all seropositive (78 case group) and a reduced number of seronegative samples (66 control group), chosen from the same farm and sampled on the same day of cases, have been genotyped by Illumina Ovine SNP600K BeadChip array, which contains about 600,000 markers covering the whole ovine genome (Illumina, San Diego, California, United States). Markers positions and names were updated to the ARS-UI_Ramb_v2.0 assembly to retain those mapped on autosomal chromosomes only. The quality control was performed on both animals and variants using PLINK v1.9 (Chang et al., 2015) with the following criteria: missing genotypes rate lower than 2%, SNP call rate larger than 98%, minor allele frequency (MAF) > 0.05. Moreover, SNPs without Hardy-Weinberg equilibrium (*p*-value<0.001) were also discarded.

### 2.4 Genome-wide analyses

Pairwise identity-by-state distances among individuals were computed using PLINK v1.9 (Chang et al., 2015) and represented by multidimensional scaling analysis (MDS).

Univariate case/control GWAS was performed by the log-additive genetic model, implemented in the SNPassoc R package (González et al., 2007), considering the herd effect. The genome-wide significant threshold was fixed according to the Bonferroni correction method as 0.05/N (N is the number of tested SNPs). GenABEL (Aulchenko et al., 2007) was used to get the genomic inflation factor (λ) and quantile-quantile (Q-Q) plot to evaluate any potential systematic bias resulting from the population structure or analytical strategy. The *p*-value and the position of each SNP along the 26 *Ovis aries* chromosomes (OAR) were reported in the Manhattan plots.

PLINK v1.9 (Chang et al., 2015) was used to perform the genome-wide F_ST_ case-control analysis. SNPs were flagged as significant if their value was larger than the 0.9999 percentile (Mastrangelo et al., 2019). Finally, Fisher’s exact test was carried out through PLINK v1.9 (Chang et al., 2015). The potential bias from multiple testing was accounted by using the Bonferroni correction (*p* < 0.05, genome-wide).

A standard descriptive Linkage disequilibrium (LD) parameter, the squared correlation coefficient of allele frequencies at a pair of loci (*r* ^2^), was obtained using PLINK v1.9 (Chang et al., 2015). The *r* ^2^ value between significant SNPs was calculated. Finally, the genotypic frequencies were also estimated.

Candidate genes close to the significant markers were retrieved using the NCBI Genome Data Viewer (https://www.ncbi.nlm.nih.gov/genome/gdv/browser/genome/?id=GCF_016772045.1). The biological functions and phenotypes associated to the annotated genes were discovered through a comprehensive literature search.

## 3 Results

On a total of 300 sheep, the results of ELISA analysis identified 78 seropositive animals, which correspond to a seroprevalence of 26%. All these samples were genotyped for the amino acid substitution at position 35 of *TMEM154*. No homozygous animals for the resistant KK genotype were observed. The most frequent genotype was EE (75% of total animals), followed by EK (25% of total animals).

Subsequently, 78 seropositive animals (case group) and other 66 seronegative animals (control group) have been genotyped using the Ovine SNP600K array. Animals of the control group were chosen from the same farms as cases and were sampled on the same day. After quality control, the final number of markers used for the analyses was 353,100, whereas the total number of individuals was 142, since two animals (control group) were discarded because of poor quality genotyping.

To examine the relationships among individuals, MDS was performed (Supplementary Figure S1). The first two components, C1 and C2, accounted for 7.6% and 5.1% of the total variation, respectively. The MDS plot did not cluster the animals according to their different phenotypes (i.e., disease status). The absence of a well-defined population configuration was confirmed by the average inflation factor (λ) of 1.28, which indicated that the population structure did not influence the results of the GWAS.

**FIGURE 1:**
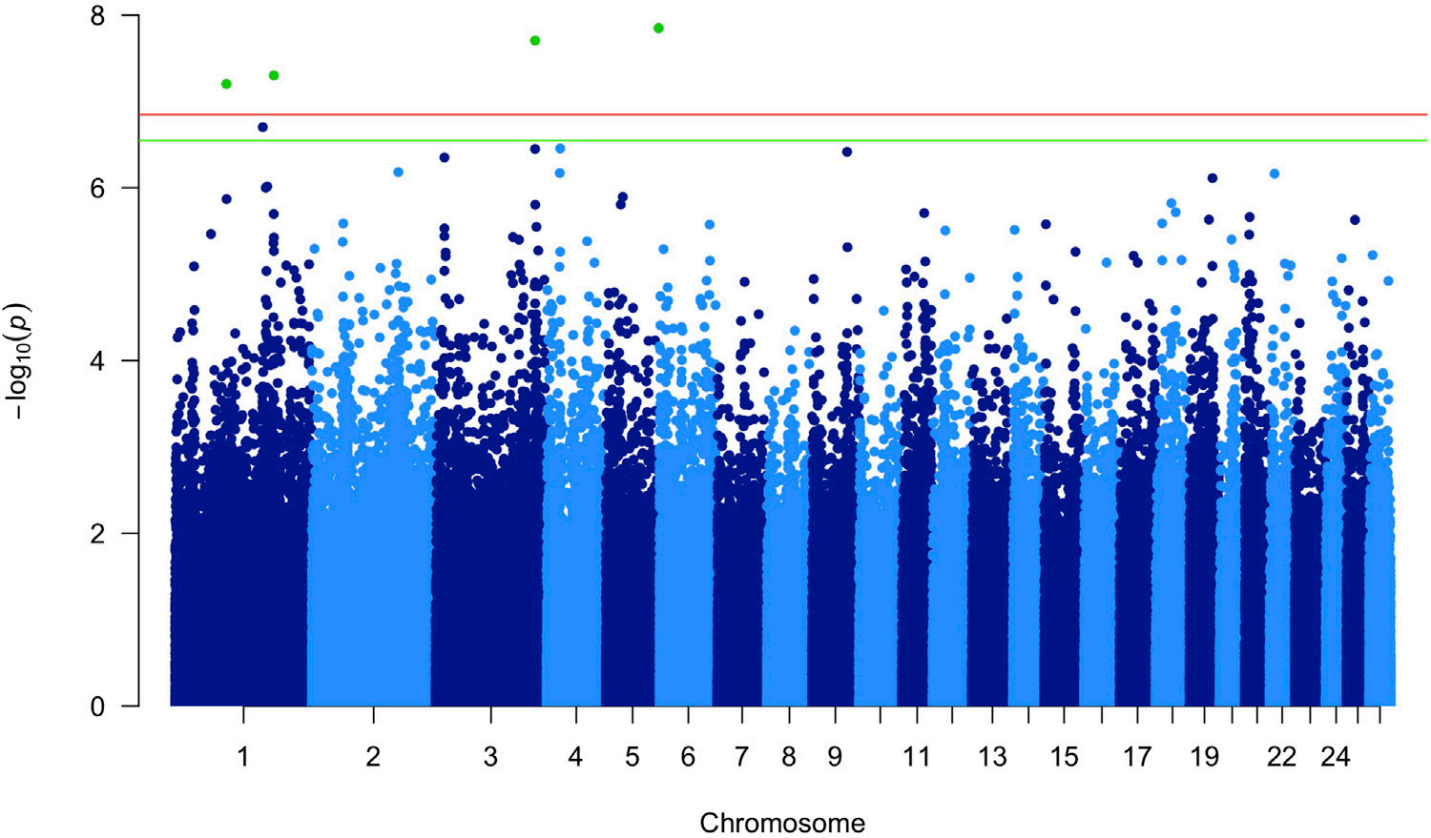
Manhattan plot of the *p*-values in the genome-wide association study (GWAS). The horizontal lines represent the genome-wide significance (red; *p* < 0.05) and suggestively significant (green; *p* < 0.10) single nucleotide polymorphisms (SNPs).

In the latter, at the *p* < 0.05 Bonferroni corrected [−log10(P) = 6.85; Pnominal value = 1.42E−07], four associated markers were identified on three different chromosomes (Table 1). Figure 1 shows the Manhattan plot obtained in this GWAS.

**TABLE 1:** Significant single nucleotide polymorphisms (SNPs) obtained using the genome-wide approaches (GWAS, Fisher’s exact test and the F_ST_ analysis) in the Valle del Belice sheep breed.

OAR	SNP name	Position bp	GWAS (*p*-value)	Fisher test (*p*-value)	F_ST_ value	Gene
1	rs418213918	104,656,798	6.273471e-08			
1	rs413444240	201,559,446	4.998098e-08		0.166	
3	rs399661659	201,728,143			0.160	
3	**rs406747332**	201,785,779	1.969503e-08	0.011	0.196	*GRIN2B*
4	rs418675045	27,413,900			0.159	
5	**rs402241447**	106,844,551	1.413548e-08	0.009	0.191	*TMEM232*
9	rs427520206	71,220,630			0.166	

Bp, base pair; GWAS, genome-wide association study; OAR, *ovis aries* chromosome number; SNP, single nucleotide polymorphism. The annotated gene is reported only for the SNPs (in bold) for which both P and F_ST_, values exceeded the indicated threshold.

The Fisher’s exact test case-control association analysis identified only two of the four significant markers found with the GWAS: rs406747332 on OAR03 and rs402241447 on OAR05 (Table 1). To support these results, a genome-wide F_ST_ case-control analysis was also performed. The analysis showed a total of six SNPs above the selected threshold (F_ST_ = 0.160), located on five different chromosomes.

The two markers with the highest F_ST_ values (>0.190) between groups overlapped with the most significant SNP_S_ identified in the GWAS [rs406747332 (−log10(P) = 7.71] and rs402241447 [−log10(P) = 7.85] (Table 1) and with the Fisher’s exact test (Table 1).

According to the LD analysis results, these two significant SNPs showed low *r* ^2^ value (0.117). The genotypic frequencies of these two SNPs exhibited variations between the two groups (Supplementary Table S1). For example, for rs406747332, the seropositive animals had a higher frequency of CC homozygous genotype (49%) than seronegative individuals, mostly heterozygous (55%). A total of two candidate genes (Table 1) were found in the surroundings (200 kb upstream and 200 kb downstream) of the markers identified with the three approaches.

## 4 Discussion

In this study, genome-wide approaches have been used to identify candidate genomic regions potentially involved with the resistance to VMV infection in the Valle del Belice sheep breed.

The serological tests showed an average seroprevalence of 26% in the Valle del Belice sheep breed. The seroprevalence presence is an important aspect, since that in herds with very low prevalence values, negative animals could be false negative due to their lower probability of infection. Consistent with our results, previous studies reported similar seroprevalence in other Italian sheep breeds (Moretti et al., 2022; Tumino et al., 2022).

The genetic involvement in susceptibility to VMV has been suggested by the breed differences reported in the literature. However, mutations in the gene *TMEM154* have been consistently associated with VMV susceptibility (Heaton et al., 2012); unfortunately, those mutations are not associated with complete resistance. White et al. (2012) conducted a GWAS on various sheep breeds, validating the findings concerning the *TMEM154* gene. Additionally, the authors identified twelve other genomic regions that could potentially be linked to the infection. Recently, Letko et al. (2021) reported no association between genotypes at the *TMEM154* gene and VMV susceptibility in the Valais Blacknose sheep, showing that this gene does not affect individual susceptibility to infection in this breed. Also, Ramírez et al. (2021) suggested that the relationship between *TMEM154* E35K genotyping and SRLV susceptibility patterns could be breed-specific and reported a positive association only in one of the four investigated sheep breeds. In addition, the high frequency of serologically positive animals (55%) carrying the less susceptible genotype KK in the Merinoland breed reported by Molaee et al. (2019) implies that the correlation between the *TMEM154* E35K and disease susceptibility in sheep needs to be approached with caution. This suggests the need for further studies to identify other genetic factors involved in developing VMV infection. Indeed, this is the first genome-wide study using a high-density SNP array aimed to identify additional candidate genomic regions potentially involved with susceptibility to VMV infection in an Italian sheep breed.

Unravelling the genetic basis of a complex trait, such as the resistance to diseases, requires careful and rigorous analysis. Based on the increased analytical power in reducing the number of false positive signals when multiple methodologies are adopted in parallel (Mastrangelo et al., 2018), this study used three distinct approaches in conjunction with the use of high-density SNP data. Despite the moderate sample size of our study, like previous GWASs in small ruminants (Mastrangelo et al., 2018; Cecchi et al., 2019; Lei et al., 2020; Letko et al., 2021; Minozzi et al., 2023), this combined approach can boost the detection accuracy and reduce unknown biases (Schiavo et al., 2018). The identification of candidate genomic regions by more than one methodology may be seen as strong evidence of the activity on a particular phenotype. This also allowed to exclude other regions that reached or were close to the defined thresholds derived by several factors that could not be better managed (i.e., genetic drift, population structure, ascertain bias of the SNP chip tool) (Schiavo et al., 2018). Therefore, the present study is sufficiently powered to find genome-wide significant SNPs related with VMV susceptibility under the hypothesis that we had ewes sampled from the same breed and there was no phenotype misclassification. Among positive and negative serological individuals, no homozygous resistant KK genotypes at the *TMEM154* gene were identified in the breed. Therefore, on the analyzed sample, this gene would not seem to affect the individual susceptibility to infection.

The results from the three different approaches highlighted the presence of shared genomic regions on OAR03 and OAR05 that are potentially associated with the observed phenotypic differences (Table 1). In fact, only two SNPs were identified associated with the analyzed trait and genome-wide approaches with few outlier markers, and in the absence of other signals, can be considered as biologically relevant (Kijas et al., 2013; Mastrangelo et al., 2019). Linkage disequilibrium between the two SNPs showed a moderate value (∼0.1). This agreed with previous studies that reported from moderate to small *r* ^2^ value for non-syntenic pairs. For example, in cattle, several authors noted that all *r* ^2^ values for non-syntenic pairs were very small (generally a low percentage has a *r* ^2^ value of more than 0.1) (Khatkar et al., 2008; Beghain et al., 2013).

One of the two significant markers (rs402241447) was located near the *GRIN2B* gene, whereas the other SNP located on OAR05 (rs402241447) was close to the *TMEM232* gene. These markers were not identified in previous studies as associated with the investigated trait. However, there is some correspondence between the results of this study and previous GWAS in sheep, as the best-associated SNP on chromosome nine at 69843937 bp, identified by Letko et al. (2021) was near to one of the six markers identified in this study using F_ST_ analysis (Table 1). The *GRIN2B* gene plays a role in receptor clustering, synaptic transmission, and pain sensitivity. The protein encoded by this gene is a NMDA receptor channel subunit for neuronal communication (Paoletti et al., 2013). Hu et al. (2016) identified the *GRIN2B* gene as potentially involved in the neurodevelopmental disorders in humans. In livestock, this gene is associated with regulating calcium channels, which are essential for several biological functions, including vasoconstriction (Witt and Huerta-Sánchez, 2019). An epigenome-wide association research found that the differentially methylated areas of the *TMEM232* gene are correlated with multiple sclerosis in humans (Souren et al., 2019). Changes in the same gene have also been associated with allergic rhinitis (Ramasamy et al., 2011). A possible involvement of the *TMEM232* gene in the development of immune diseases has also been suggested (Han et al., 2023). In cattle, it was reported as a differentially expressed gene between lactation stages (Bhat et al., 2019), and involved in behavior traits (Friedrich et al., 2016). To our knowledge, these genes have not previously been associated with lentiviral infection in any species. In a previous GWAS, White et al. (2012) reported different candidate genes, but as in our study, those genes were not previously identified. After all, before the study of Heaton et al. (2012), even the function of the major gene *TMEM154* had never been reported for any species. In fact, a previous study identified *TMEM154* in a human GWAS for asthma severity (Slager et al., 2011), suggesting that this gene may play a role in immune responses, with a function similar to the *TMEM232* here identified as a new potential candidate gene. Moreover, it is noteworthy that both genes belonging to the TMEM family, code for proteins that are predicted to be components of diverse cell membranes, such as mitochondrial, endoplasmic reticulum, lysosome, and Golgi membranes. TMEMs are widely present across different cell types and play key roles in essential functions such as epidermal keratinization, autophagy, and smooth muscle contraction. Additionally, certain TMEM proteins are critical in immune response, and they are key components of inflammatory signaling pathways, facilitating the production of pro-inflammatory cytokines (Schmit and Michiels, 2018).

## 5 Conclusion

Genetic selection of resistant animals is of particular interest in viral diseases lacking effective treatments or vaccines. Our study suggests new aspects of the genetic complexity related to the resistance/susceptibility to SRLV in sheep species. Moreover, it describes for the first time the involvement in susceptibility/resistance to VMV of some genes never identified before, confirming that studies on different breeds can lead to different results. The ideal approach for validation of the markers identified in our study is to use samples from a population independent from the discovery population with the same phenotype used in the discovery stage. Additional analysis based on sequencing and increased number of genotyped sheep would help confirm our findings, refine these results, and better understand the genetic basis of VMV.

## Data Availability

The data presented in the study are deposited in the figshare repository, accession number 10.6084/m9.figshare.25858267. This data can be found here: https://doi.org/10.6084/m9.figshare.25858267.v1

## Supplementary material

Supplementary Table S1 Genotypic frequencies of the best associated markers for case/control samples.

Supplementary Figure S2 Multidimensional scaling (MDS) plot of seropositive (red) and seronegative (blue) individuals.
