# Supplementary material for: A high-density genome-wide approach reveals novel genetic markers linked to small ruminant lentivirus susceptibility in sheep
Source: Front Genet. 2024 Jun 6;15:1376883. doi: 10.3389/fgene.2024.1376883 (PMC11191640; doi:10.3389/fgene.2024.1376883)
Supplement: Supplementary file 1 [file Table1.DOCX]

A high-density genome-wide approach reveals novel genetic markers linked to small ruminant lentivirus susceptibility in sheep

Silvia Riggio^1^, Marco Tolone^2^, Gianluca Sottile^3^, Serena Tumino^4^, Baldassare Portolano^1^, Anna Maria Sutera^2^, Maria Teresa Sardina^1^, Alberto Cesarani^5,6^, Salvatore Mastrangelo^1^

***Supplementary Material***

**Supplementary Figure 1.** Multidimensional scaling (MDS) plot of seropositive (red) and seronegative (blue) individuals.

Supplementary Table S1 Genotypic frequencies of the best associated markers for case/control samples.

| **SNP name** | **Group** | **Genotype** | | |
| --- | --- | --- | --- | --- |
| rs406747332 | Case | AA (0.18) | AC (0.33) | CC (0.49) |
|  | Control | AA (0.41) | AC (0.55) | CC ( 0.04) |
| rs402241447 | Case | AA (0.01) | AG (0.22) | GG (0.77) |
|  | Control | AA (0.19) | AG (0.45) | GG (0.36) |
